# Profiling Cullin4-E3 Ligases Interactomes and Their Rewiring in Influenza A Virus Infection

**DOI:** 10.1016/j.mcpro.2024.100856

**Published:** 2024-10-09

**Authors:** Guillaume Dugied, Thibaut Douche, Melanie dos Santos, Quentin Giai Gianetto.Q, Camille Cassonnet, Françoise Vuillier, Patricia Cassonnet, Yves Jacob, Sylvie van der Werf, Anastassia Komarova, Mariette Matondo, Marwah Karim, Caroline Demeret

**Affiliations:** 1 Unit of Molecular Genetics of RNA Viruses, Institut Pasteur, Paris, France; 2 Interactomics, RNA and Immunity Laboratory, Institut Pasteur, Paris, France; 3 Institut Pasteur, Proteomics Core Facility, MSBio UtechS, UAR CNRS 2024, Université Paris Cité, Paris, France; 4 Institut Pasteur, Bioinformatics and Biostatistics Hub, Université Paris Cité, Paris, France

**Keywords:** influenza A virus, Cullin 4-RING E3 ubiquitin ligase, affinity purification coupled with mass spectrometry, protein-protein interactions, split-nanoluciferase assay

## Abstract

Understanding the integrated regulation of cellular processes during viral infection is crucial for developing host-targeted approaches. We have previously reported that an optimal *in vitro* infection by influenza A virus (IAV) requires three components of Cullin 4-RING E3 ubiquitin ligases (CRL4) complexes, namely the DDB1 adaptor and two substrate recognition factors, DCAF11 and DCAF12L1, which mediate non-degradative poly-ubiquitination of the PB2 subunit of the viral polymerase. However, the impact of IAV infection on the CRL4 interactome remains elusive. Here, using Affinity Purification coupled with Mass Spectrometry (AP-MS) approaches, we identified cellular proteins interacting with these CRL4 components in IAV-infected and non-infected contexts. IAV infection induces significant modulations in protein interactions, resulting in a global loss of DDB1 and DCAF11 interactions, and an increase in DCAF12L1-associated proteins. The distinct rewiring of CRL4’s associations upon infection impacted cellular proteins involved in protein folding, ubiquitination, translation, splicing, and stress responses. Using a split-nanoluciferase-based assay, we identified direct partners of CRL4 components and via siRNA-mediated silencing validated their role in IAV infection, representing potential substrates or regulators of CRL4 complexes. Our findings unravel the dynamic remodeling of the proteomic landscape of CRL4’s E3 ubiquitin ligases during IAV infection, likely involved in shaping a cellular environment conducive to viral replication and offer potential for the exploration of future host-targeted antiviral therapeutic strategies.

## Highlights

• IAV infection leads to a remodeling of the CRL4 E3 ubiquitin ligase interactome.
• Distinct CRL4 factors target different cellular processes and stress responses in IAV.
• CRL4 interactors with a role in IAV infection highlight antiviral and proviral targets.

## Graphical Abstract

**Figure:**
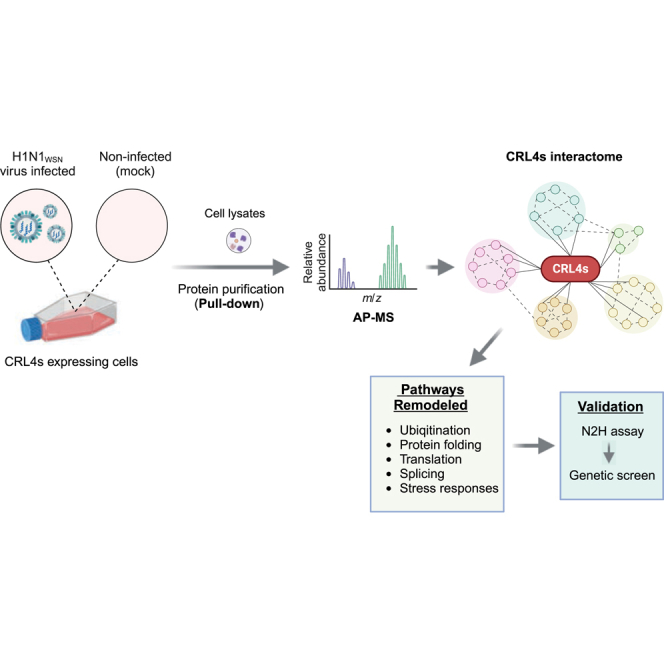

## In Brief

Our study reveals how influenza A virus (IAV) infection rewires the CRL4 E3 ubiquitin ligase complex’s interactome. Using Affinity Purification coupled with Mass Spectrometry (AP-MS), we show that IAV infection disrupts interactions of CRL4 components, including DDB1 and DCAF11, while enhancing DCAF12L1 associations. This dynamic remodeling impacts **proteins** crucial for cellular processes including protein folding, ubiquitination, and stress responses. Our findings highlight potential targets for novel host-directed antiviral therapies by detailing the altered functional landscape of CRL4s during infection.

Viruses co-opt the host cellular machinery by engaging in protein-protein interactions with the host proteome to facilitate their replication and overcome cellular restrictions. Ubiquitination, a major post-translational modification, involves the covalent attachment of ubiquitin molecules to target proteins, which can be reversed by deubiquitination (1). Ubiquitination is mediated by an enzymatic cascade involving ubiquitin activation (E1 ubiquitin-activating enzymes), conjugation (E2 ubiquitin-conjugating enzymes), and transfer (E3 ubiquitin ligases responsible for the recruitment of substrate proteins), while ubiquitin removal is mediated by de-ubiquitinases (DUBs). Multiple types of protein ubiquitination exist, such as mono-ubiquitination and poly-ubiquitination, wherein variations in ubiquitin linkages depend on the lysine residue engaged in poly-ubiquitin chain formation (2, 3). The specific type of ubiquitin chains determines the fate of the protein substrate, i.e., proteasome-mediated degradation, regulation of signaling pathways, or localization to specific subcellular compartments. The targeting of ubiquitinated proteins to the proteasome for degradation is a major pathway of regulated protein stability, and ubiquitination plays a central role in multiple cellular processes, such as cell cycle progression, signal transduction, antigen presentation, and immune responses (4).

The set of proteins driving ubiquitination and its regulation, termed the Ubiquitin-Proteasome System (UPS), plays a dual role in viral pathogenesis, manifesting both pro-viral and antiviral effects. Indeed, the UPS can enhance viral protein function by inducing their non-degradative ubiquitination or modifying host factors required for the viral life cycle (5). Conversely, it can act as a restriction factor by promoting ubiquitin-dependent degradation of viral proteins, activating immune signaling pathways, or controlling cell cycle events (6). This dual role makes the UPS a key target for viruses to rewire the degradative apparatus and manipulate host signaling pathways (7, 8). From a molecular standpoint, virus-induced rewiring of E3-ligases results from either gaining interactions via the recruitment of new protein partners or losing interactions with natural cellular protein substrates. In the context of influenza A virus (IAV) infection, the involvement of the UPS has been documented at various stages of the viral life cycle, resulting in both pro- and anti-viral outcomes (9). For instance, NS1 protein prevents RIG-I ubiquitination by interacting with TRIM25 E3 ligase, suppressing the antiviral interferon response (10), while ubiquitination of matrix protein M1 by Itchy E3 ligase aids the release of viral genomic segments from late-endosomes (11). Ubiquitination of polymerase subunits PB1, PB2, PA, and nucleoprotein (NP) contributes to viral genome replication and transcription (12, 13). Furthermore, ubiquitination of NP by the CNOT4 E3 ligase promotes viral replication (14), while polyubiquitination by TRIM22 and TRIM41 E3 ligases targets NP for proteasome-dependent degradation (15, 16), a process reversed by the de-ubiquitinase USP11 (17).

We have previously demonstrated that the PB2 protein of IAV undergoes non-degradative ubiquitination at different lysines, mediated by two RING-E3 ubiquitin ligases based on Cullin 4 (CRL4s), thereby promoting the viral life cycle (18). PB2 directly binds to the recruitment modules of these CRL4s, consisting of the DDB1 adaptor and the substrate recognition factors (SRF) DCAF11 (CRL4^DCAF11^) or DCAF12L1 (CRL4^DCAF12L1^) (19). Several viral encoded proteins have been reported to hijack CRL4s E3 ligases for ubiquitination, leading to the degradation of antiviral or regulatory host factors by bridging a cellular protein to DDB1 or to an SRF (20, 21, 22). While this infection-induced rewiring of E3 ubiquitin ligases is a recurring strategy of viruses for tailoring the cellular environment to their replication cycle, such phenomena have never been documented for IAV infection. To bridge this gap, we investigated whether the substrate specificity of these CRL4s is altered during infection, using affinity purification-mass spectrometry (AP-MS). Our results revealed substantial modulations in the interaction networks of the three factors, DDB1, DCAF11, and DCAF12L1, characterized by a comprehensive reduction in DDB1 and DCAF11 interactions, while DCAF12L1 interactors increased upon infection. Furthermore, we assessed a subset of these interactors for their direct interactions with the CRL4s factors using a mammalian cell-based nano-luciferase (N2H) assay, unveiling potential infection-regulate substrates or regulators of these E3 ligases. The silencing of a sub-set of CRL4s targets showed their involvement in IAV infection. These findings unravel the proteomic landscape entwined with CRL4's E3 ubiquitin ligases and their dynamic remodeling during IAV infection and provide a foundation for future exploration of host-targeted antiviral therapeutic strategies.

## Experimental Procedures

### Cell Culture and Generation of Stable Cell Lines

Human embryonic kidney cell lines HEK-293T and HEK-293 (ATCC) were cultured in complete Dulbecco’s modified Eagle’s medium (DMEM) supplemented with 10% v/v fetal calf serum (FCS) and 1% Pen-strep antibiotics. Canine Kidney-derived MDCK-SIAT cells (23) were grown in Modified Eagle's Medium (MEM) supplemented with 5% FCS.

For the generation of stable cell lines, HEK293 cells were transfected using Jetprime (Polypus) with expression vectors for Strep-tagged DDB1, DCAF11, DCAF12L1, or mCherry. At 48 h post-transfection, cells were selected for neomycin (400 μg/ml) resistance. Single clones were sorted, expanded, and assessed for Strep fusion protein expression levels by immunoblotting. All cells were maintained in a humidified incubator with 5% CO_2_ at 37 °C and tested negative for *mycoplasma* using the MycoAlert *mycoplasma* detection kit (Lonza).

### Viruses and Infection

The recombinant influenza virus strain A/WSN/33 (H1N1_WSN_) was produced by reverse genetics, as mentioned in the reference (24). For infection, HEK293 cells stably expressing Strep-tagged CRL4 factors and mCherry were mock-treated medium alone or infected with the influenza virus strain A/WSN/33 (H1N1_WSN_) at a multiplicity of infection (MOI) of 3 for 6 h at 37 °C.

### Plasmids

For protein complementation assay based on the split luciferase (N2H) assay, the Gateway-entry plasmids containing the DDB1, DCAF11, DCAF12L1, and other cellular proteins ORFs were obtained from the human ORFeome resource (Center for Cancer Systems Biology (CCSB) human ORFeome 8.1 collection). To generate vectors encoding complementary NanoLuc fragments F1- and F2-, as well as Strep-fusion proteins, the ORFs were transferred into a Gateway compatible pDESTN2H-GW or pIBA105-GW destination vectors using recombination cloning (Gateway technology, Invitrogen).

### Gene-specific RT-qPCR Assay

HEK293 cells stably expressing Strep-tagged CRL4 factors or A549-ACE2 cells depleted for CRL4s interactors were harvested, lysed, and subjected to total RNA extraction with the RNeasy mini-kit (Qiagen). RT-qPCR was conducted with gene-specific forward and reverse primers, following the manufacturer’s protocol outlined in the LightCycler RNA amplification kit SYBR green I (Roche). The sequences of the oligo couple will be provided upon request. The cellular glyceraldehyde-3-phosphate dehydrogenase (GAPDH) mRNA in the infected cells served as an internal control.

### Strep Pulldown and Western Blot

HEK293 cells stably expressing Strep-tagged CRL4 factors or mCherry were washed twice with cold PBS and lysed in 20 mM MOPS-KOH (pH 7.4), 120 mM KCl, 2 mM ß-mercaptoethanol, 0.5% IGEPAL supplemented with 1× protease inhibitor Cocktail (Roche) for 30 min on ice. For Strep-tag purification, cell lysates were clarified by centrifugation at 16,000*g* for 15 min at 4 °C and incubated with Strep-Tactin beads (Strep-Tactin Sepharose high performance; GE Healthcare) for 2 h at 4 °C on a spinning wheel. Beads were washed three times in wash buffer (20 mM MOPS-KOH, pH 7.4, 120 mM of KCl, 2 mM β-mercaptoethanol, protease inhibitor cocktail).

For Western blot analysis, the bound protein complexes were eluted from Strep-Tactin beads with desthiobiotin and diluted in Laemmli buffer (Invitrogen). Immunoblot membranes were incubated with peroxidase-conjugated Strep-Tactin or with primary antibody directed against PB2 (GTX125926, GeneTex), then an HRP-conjugated anti-rabbit antibody (GE Healthcare), then revealed with the ECL2 substrate (Pierce). The chemiluminescence signals were acquired using a G-Box and the GeneSnap software (SynGene).

### Silver Staining and Coomassie Staining

After Strep pulldown, protein complexes were eluted from Strep-Tactin beads with desthiobiotin. Eluted proteins were precipitated using 12% TCA and washed with ice-cold acetone. The purified proteins were analyzed by SDS-PAGE and revealed by silver staining (Sigma) according to the manufacturer's guidelines.

For Coomassie blue staining, the experiment was performed as described above. The whole cell lysates (WHL), flow-through after immuno-precipitation, washes 1, 2, and 3, beads after elution of protein, and the eluted proteins after TCA precipitation were loaded in the SDS-PAGE and revealed by instant blue Coomassie staining (Expedeon).

## Sample Preparation for Mass Spectrometry

### Pull-Down Digestion

After Strep pulldown, protein complexes were eluted from Strep-Tactin beads with desthiobiotin. Eluted proteins were precipitated using 12% TCA (final concentration) overnight at 4 °C. Protein pellets were washed twice with ice-cold acetone and then resuspended in a denaturation buffer containing 8M urea and 100 mM NH_4_HCO_3_. Cysteine bonds were reduced with 50 mM TCEP (Sigma-Aldrich) for 1 h and sonicated twice for 1 min each on ice. Proteins were alkylated with 50 mM iodoacetamide (Sigma-Aldrich) for 1 h at room temperature in the dark. Samples were digested with rLys-C (Promega) ratio 50:1 (protein:rLysC) for 4 h at 37 °C and then digested with Sequencing Grade Modified Trypsin (Promega, France) ratio 25:1 (protein: trypsin) overnight at 37 °C after a dilution in 100 mM NH_4_HCO_3_ to decrease the urea under 1M. The digestion was stopped with 4% formic acid (FA) (Fluka), and digested peptides were purified with C18 Spin Columns Pierce (ThermoFisher Scientific). Peptides were eluted with 2 × 80% Acetonitrile (ACN)/0.1% FA. The resulting peptides were speed-vac dried and resuspended in 2% ACN/0.1% FA for further analysis.

### LC-MS/MS

LC-MS/MS analysis of CRL4s–specific protein complexes was performed on a Q Exactive Plus Mass Spectrometer (Thermo Fisher Scientific) coupled with a Proxeon EASY-nLC 1000 (Thermo Fisher Scientific). The same volume of peptides was injected into a homemade 35-cm C18 column (1.9-μm particles, 100-Å pore size, ReproSil-Pur Basic C18, Dr Maisch GmbH, Ammerbuch-Entringen) and eluted with a multistep gradient from 2 to 7% buffer B (80% ACN/0.1% FA) for 5 min, seven to 23% for 70 min, 23 to 45% for 30 min, and 45 to 95% fir 5 min, at a flow rate of 250 nl/min over 132 min. The column temperature was set to 60 °C. MS data were acquired using Xcalibur software using a data-dependent method. MS scans were acquired at a resolution of 70,000, and MS/MS scans (fixed first mass 100 m/z) at a resolution of 17,500. The automatic gain control (AGC) target and maximum injection time for the survey scans and the MS/MS scans were set to 3 × 10^6^, 20 ms, and 1 × 10^6^, 60 ms, respectively. An automatic selection of the 10 most intense precursor ions was activated (top 10) with a 45-s dynamic exclusion. The isolation window was set to 1.6 m/z and normalized collision energy fixed to 28 for higher energy collisional dissociation fragmentation. We used an underfill ratio of 1.0% for an intensity threshold of 1.7 × 10^5^. Unassigned precursor ion charge states, as well as 1, 7, 8, and >8 charged states, were rejected, and peptide match was disabled.

### Bioinformatics Analysis of LC-MS/MS Data

The analysis of raw data was performed using MaxQuant software version 1.5.5.1. The MS/MS spectra were searched against two databases: the Human Swiss-Prot database (comprising 20,203 entries from the UniProt, dated 12/04/2018) and the personal Influenza A virus database (8 entries). Variable modifications (methionine oxidation, N-terminal acetylation, and lysine ubiquitinylation) and fixed modification (cysteine carbamidomethylation) were set for the search, and trypsin with a maximum of two missed cleavages was chosen for searching. The minimum peptide length was set to seven amino acids, and a false discovery rate (FDR) of 0.01 was applied for peptide and protein identification. Identification of proteins mandated at least a unique peptide per protein group. The main search peptide tolerance was set to 4.5 ppm, with the MS/MS match tolerance set at 20 ppm. Second peptides were enabled to identify co-fragmentation events, and a match between runs option was selected, with a match time window of 0.7 min for an alignment time window of 20 min. Quantification was performed using the XIC-based LFQ algorithm with the Fast LFQ mode as described previously (25). Acceptance criteria for quantification included unique and razor peptides, including modified peptides, with at least 2 ratio counts. The mass spectrometry proteomics data have been deposited to the ProteomeXchange Consortium via the PRIDE (26) partner repository with the data set identifier PXD050261.

### AP-MS Analysis Pipeline

For the differential analyses, proteins identified in the reverse and contaminant databases and proteins “only identified by site”, were first excluded from the list of identified proteins. Then, only proteins with two quantified intensity values in a given condition are kept. Following log2 transformation, LFQ values were normalized by median centering within conditions, using the normalized function of the R package DAPAR (27). The remaining proteins without any LFQ value were considered as present in one condition and absent in another. These proteins were set aside and considered as differentially abundant proteins. Next, missing values were imputed using the imp.norm function of the R package norm (28). Proteins with a fold-change under 2.0 were considered not significantly differentially abundant. For the remaining proteins with a fold-change over 2.0, statistical analysis was conducted using a limma *t* test (29), with the R package limma (30). An adaptive Benjamini-Hochberg procedure was applied to the resulting *p*-values using the function adjust.p of R package cp4p (31), employing the robust method (32), to estimate the proportion of true null hypotheses among the set of statistical tests. The proteins associated with an adjusted *p*-value below FDR of 1% were considered significantly differentially abundant. Finally, the proteins of interest are those that emerged from this statistical analysis, complemented by those that are present in one condition and absent in another.

### Experimental Design and Statistical Rationale for MS

MS experiments were conducted in three independent biological replicates to ensure the robustness and reproducibility of the results. As shown in Figure 2*A* and Supplemental Fig. S2, the correlation between each pair of samples was computed using all complete pairs of observations in these samples. A high level of confidence (>0.8) was obtained, ensuring the reliability of the findings reported in the MS-based proteomic study.

### GO Enrichment Analysis

The GO term enrichment analysis of the AP-MS datasets was performed using gProfiler (33), with database versions Ensembl 110, Ensembl Genomes 56 and Wormbase ParaSite 18. The transcriptome data of HEK293 taken from the Human Protein Atlas (version 21.1) (34) was used as the background dataset. Adjusted *p*-values were calculated using the Benjamini-Hochberg procedure. Subsequent analysis was performed using R (https://www.R-project.org/).

### Mammalian N2H Assay

Mammalian N2H assay was performed as described in Choi *et al*. (35). Briefly, HEK293T cells were seeded the day before transfection at 6 x 10^4^ cells per well in 96-well plates. Cells were then transfected with 100 ng of each N2H plasmid (pN2H-N1 or C1 plus pN2H-N2 or C2). Twenty-4 hours after transfection, 50 μl of a 100× diluted NanoLuc substrate was added per well after removal of the culture medium. The substrate used (Q-108) was obtained in a concentrated solution, from the corresponding O-acetylated derivative hikarazine-108, following acidic hydrolysis as previously described (36, 37, 38). Luciferase enzymatic activity was measured using a CentroXS luminometer (Berthold; 2 s integration time). For each protein pair, the obtained RLU was divided by the RLU generated by each of the partners co-expressed with the complementary nanoluciferase fragment expressed either unfused or fused to a non-interacting protein (commonly referred to as N1-CTRL or N2-CTRL), giving a Normalized Luminescence Ratio (NLR) as follows: NLR = RLU [N1-CellP x N2-CRL4]/RLU [N1-CellP x N2 CTRL] + [N1-CTRL x N2-CRL4]. The calculation of NLR thus enables consideration of the interaction background of each partner in the protein pairs under examination.

### siRNA Transfection and Efficiency

siRNA transfection and efficiency. siRNAs targeting the CRL4’s partners were purchased from Dharmacon (ON-TARGETplus SMARTpools and non-targeting control). A549 cells were transfected with 25 nM siRNA using the Interferin transfection reagent (Polyplus). At 48 h post-transfection, cells were infected with the A/WSN/33 (H1N1) at a multiplicity of infection (moi) of 10−3 pfu/cell. Plaque assays with MDCK cells were performed as described in (39). Viral titer is expressed as ratios relative to the titers obtained with non-targeting siRNA. Data were analyzed with GraphPad Prism using Mann-Whitney multiple comparison *t* test.

## Results

### Overview of the Methodology for Identifying CRL4 Factors Proteomes in IAV-Infected Cells

**Fig. 1:**
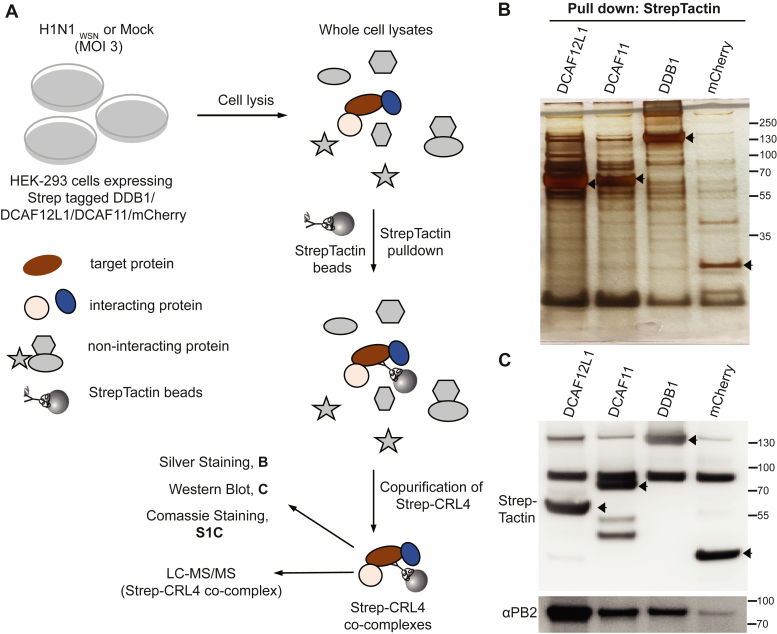
**Comprehensive identification of CRL4 co-complexes in IAV infection via Affinity Purification- Mass Spectrometry (AP-MS).***A*, schematics of the steps to purify Strep-CRL4 and associated cellular proteins from H1N1_WSN_ infected cells for MS analysis. HEK-293 cell lines stably expressing Strep-tagged fused DDB1, DCAF11, DCAF12L1, or mCherry (control) were infected with H1N1_WSN_ (MOI = 3) or mock-infected. Cells were harvested after 6 hpi, lysed, and equivalent amounts of total proteins were subjected to Strep pull down using StrepTactin beads. Purified protein complexes were eluted from the beads using a biotin elution buffer, followed by precipitation using 12% TCA. The proteins co-purifying with Strep-CRL4 factors or with Strep-mCherry were digested into peptides and analyzed by Mass spectrometry to measure changes in protein abundance upon infection compared to mCherry control and non-infected condition. Equivalent amounts of the whole cell lysates (input) were also analyzed by MS. All conditions were performed in biological triplicate. *B*, proteins co-eluted with the indicated Strep-CRL4s factors after StrepTactin-affinity purification (pull down) were visualized by silver staining of SDS gels. *C*, detection of PB2 co-purified with CRL4 factors d by Western blot. The bands corresponding to Strep-CRL4 factors and mCherry are indicated with arrows.

**Fig. 2:**
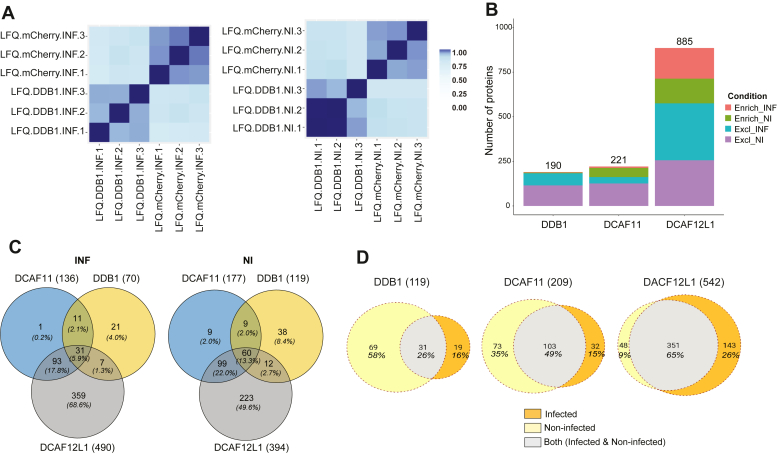
**Global comparison of the host CRL4 interactomes during H1N1**_**WSN**_**infection**. *A*, correlation matrix of the three biological replicates of HEK-293 cell lines stably expressing Strep-DDB1 versus Strep-Cherry controls in H1N1_WSN_ infected cells (*Left*) or non-infected cells (*Right*). *B*, stacked to the total number of partners found either as enriched relative to the control cherry pull-down or as exclusive (*i.e.* not detected associated with Strep-mCherry) in infected and non-infected condition (Excl_INF, Excl_NI, Enrich_INF, Enrich_NI). *C*, Venn diagrams depicting the number and percentage of interactors in infected, non-infected, or both (both) conditions are shown in *orange*, *yellow*, and *gray* respectively. *D*, Venn diagrams showing the number and percentage of common CRL4 interactors from the mass-spectrometry in the infected (*left*) and non-infected (*right*) conditions. Excl, exclusive; INF, infected; NI, non-infected.

We have previously shown that the PB2 subunit of IAV RNA-dependent RNA polymerase undergoes non-degradative poly-ubiquitination following its binding to various factors of the multi-components CRL4 E3 ligases, namely the adapter DDB1 and the SRFs, DCAF11 and DCAF12L1 (18). To assess the rewiring of these CRL4s E3 ligases upon IAV infection, we explored the landscape of the cellular proteins bound to these CRL4 components in infected and non-infected cells using Affinity Purification-Mass Spectrometry (AP-MS) (Fig. 1*A*). For this purpose, we generated HEK293 cells stably expressing each Strep-tagged CRL4 factor and Strep-mCherry as controls. A single clone of each cell line was selected based on the comparable expression levels of Strep-tagged proteins among Strep-tagged CRL4s and Strep-mCherry (Supplemental Fig. S1*A*). Assessment of DDB1 levels showed no strong overexpression of Strep-DDB1 compared with its endogenous protein, while the endogenous DCAF11 or DCAF12L1 could not be detected owing to a lack of suitable antibodies (Supplemental Fig. S1*A*). We detected a higher expression of DCAF12L1 protein and mRNA levels than DCAF11 and DDB1 (Supplemental Fig. S1*B*). We employed AP-MS analysis to investigate the landscape of cellular proteins bound to each CRL4 factor (DDB1, DCAF12L1, and DCAF11) in HEK293 cells stably expressing Strep-tagged CRL4s and Strep-mCherry (as a negative control), either mock infected or infected with H1N1_WSN_ at MOI of 3. At 6 h post-infection, cells were lysed, and a Strep pull-down was performed on an equivalent amount of whole cell lysates. The co-purifying proteins eluted from the StrepTactin-beads were identified via LC-MS/MS analysis (Fig. 1*A*). The purification of Strep-tagged proteins was monitored throughout the pull-down procedure by Coomassie blue staining (Supplemental Fig. S1*C*), and the pattern of eluted proteins was analyzed by silver staining, with prominent bands corresponding to the pulled Strep-tagged proteins (Fig. 1*B*). Immunoblot analysis of the pulled fractions from IAV-infected cells revealed PB2 co-precipitation with all three CRL4 factors, while only marginal PB2 co-purified with the Strep-mCherry control (Fig. 1*C*). It should be noted that the PB2 protein level is too low to be directly detectable in infected cell lysates before pull-down in the volume of extraction for AP-MS. PB2 has nevertheless been detected after the pull-down with the CRL4s factors (Fig. 1*C*), in line with our earlier findings in similar experimental conditions (18). For each Strep-CRL4 and Strep-mCherry expressing cell, three independent infections were performed, in parallel to mock treatment, followed by affinity chromatography purification and LC-MS/MS analysis. Each series of biological replicates exhibited a high number of consistently quantified proteins/peptides, strongly correlated, and consistent distribution across all replicates indicating robust experimental reproducibility and reliability of our quantification process (Fig. 2*A*, Supplemental Fig. S2, *A–F* and Supplemental Table S1). Following quality control measures and in-house statistical pipelines, proteins detected in at least two replicates were retained for each condition (see Experimental Procedures). The overall protein levels quantified in inputs between the three CRL4s are comparable across all cell extracts, regardless of the infection status (Supplemental Table S1). Proteins present only in Strep-CRL4-expressing but not in Strep-mCherry cells, were assigned as exclusively associated with the corresponding CRL4 factor.

For the remaining non-exclusive proteins, a differential analysis was applied to identify those significantly more abundant with the CRL4 factors than with the mCherry control (log2 FC over mCherry >1, *p*-value < 0.001). Our AP-MS analysis identified a total number of 190, 221, and 885 high confidence (HC) interactions with DDB1, DCAF11, and DCAF12L1, respectively, combining infected and non-infected conditions, with a constant higher proportion of proteins exclusively associated with the CRL4s factors, indicating a correct stringency of the pull-down (Fig. 2*B* and Supplemental Table S1). The higher number of proteins detected for DCAF12L1 is likely attributable to a higher accumulation of Strep-DCAF12L1, which was observed by silver staining of the eluted proteins after Strep-DCAF12L1 pull-down and despite our efforts to minimize differences in CRL4 expression levels (Fig. 1*B*). Combining infected and non-infected conditions, 119, 209, and 542 cellular proteins were identified associated with DDB1, DCAF11, and DCAF12L1 (Fig. 2*C* and Supplemental Table S2). A significant rewiring of the CRL4s interaction partners was observed, particularly for DDB1 for which only 26% of the 119 interactors remained constant upon infection, whereas 49% and 65% remained unchanged for DCAF11 and DCAF12L1, respectively. A global loss of interacting protein partners during influenza infection is observed for both DDB1 and DCAF11, which interact respectively with 69 and 73 proteins only in non-infected cells, and with 19 and 33 exclusively in infected cells (Fig. 2*C* and Supplemental Table S2). Conversely, DCAF12L1 gains cellular interactors upon infection, with 143 only in infected cells compared to 48 proteins associated exclusively in non-infected cells (Fig. 2*C*). Among the HC interactors, 60 were identified to interact with all CRL4s in the non-infected condition, against 31 in the infected condition (Fig. 2*D* and Supplemental Table S2). Additionally, we observed an increased proportion of factors interacting only with DCAF12L1 in the infection compared to the non-infected condition.

### The DDB1 Interactome During IAV Infection

**Fig. 3:**
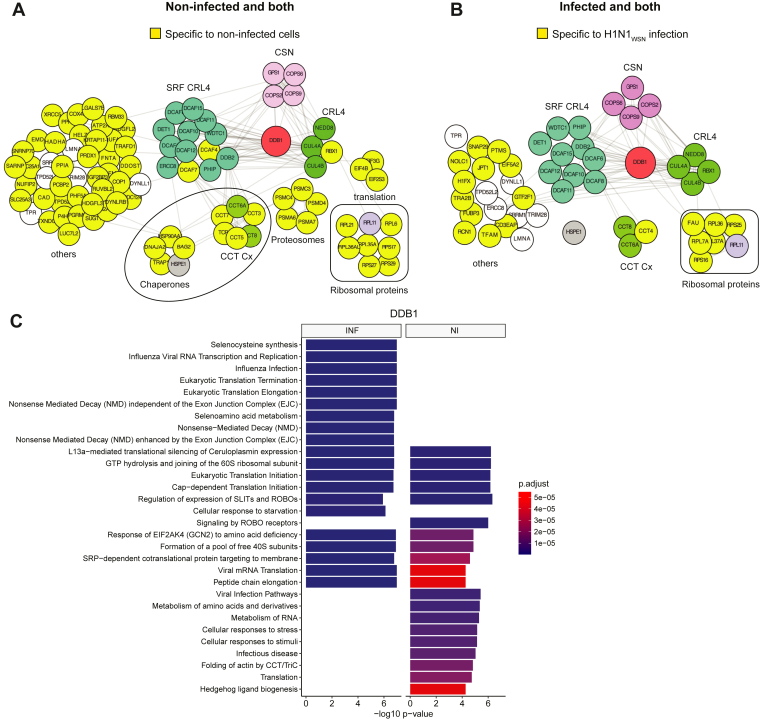
**Protein interaction network of DDB1-associated proteins upon H1N1**_**WSN**_**infection.***A*, proteins co-purified with DDB1 in non-infected cells, corresponding to the combination of proteins associated with DDB1 in both non-infected and infected conditions (both) and specifically those found in non-infected cells. *B*, proteins co-purified with DDB1 in infected cells, correspond to the combination of proteins identified in both conditions and those only in infected cells. Clusters of proteins functionally enriched are highlighted in different colors. Proteins associated with DDB1 specifically in non-infected (*A*), or infected (*B*) conditions are depicted in *yellow*. *C*, gene Ontology enrichment analysis of the significantly altered proteins using Benjamini-Hochberg correction analysis. HEK293 gene set from the Human Protein Atlas was used as a reference for background. INF= H1N1_WSN_ infected condition, NI = non-infected condition. All DDB1-associated proteins are listed in Supplemental Table S2.

DDB1 is a well-characterized protein, and several of its interactors have previously been identified by mass spectrometry in non-infectious conditions (40, 41). We detected numerous known interacting proteins, including Cul4 (A and B), RBX1, and several DWD-containing substrate recognition receptors (DCAFs), underscoring the presence of physiological DDB1 partners related to its role as an adapter of CRL4 E3 ligases. Additionally, members of the COP9 signalosome complex (CSN) were detected in association with DDB1, in line with its role in regulating CRL4 E3 ligases (Fig. 3*A*). The association of DDB1 with the CRL4s-related factors essentially remained unchanged upon infection. Furthermore, we identified several proteasome subunits, indicating the delivery of some CRL4s E3 ligases targets for proteasome-mediated degradation, specifically in non-infected cells. Beyond these known interactions, our approach detected alterations in the recruitment of cellular proteins to DDB1, including translation regulators, ribosomal proteins, chaperones, and members of the Chaperonin Containing TCP-1 (CCT) complex. DBB1 was strongly remodeled upon IAV infection, with the majority of these changes being specific to non-infected cells (Fig. 3, *A* and *B*).

We performed a pathway enrichment analysis by querying the REACTOME database with the list of factors gained or lost upon infection (Supplemental Table S3). By comparing the top 20 enriched pathways in both lists of factors, we identified pathways related to influenza infection, influenza virus RNA transcription, and replication as amongst the most significant pathways enriched upon infection, suggesting a shift of DDB1-associated factors toward factors involved in influenza virus infection (Fig. 3*C*). Infection-specific DDB1-associated factors targeted translation elongation and termination as well (Fig. 3*C*). Pathways related to virus infection and mRNA translation, metabolisms of amino acids, RNA and cellular responses to stress/stimuli were enriched in non-infected cells, suggesting their potential hijacking by the virus during infection.

### Substrate Recognition Receptors DCAF11 and DCAF12L1 Exhibit Distinct Protein Associations in Response to IAV Infection

**Fig. 4:**
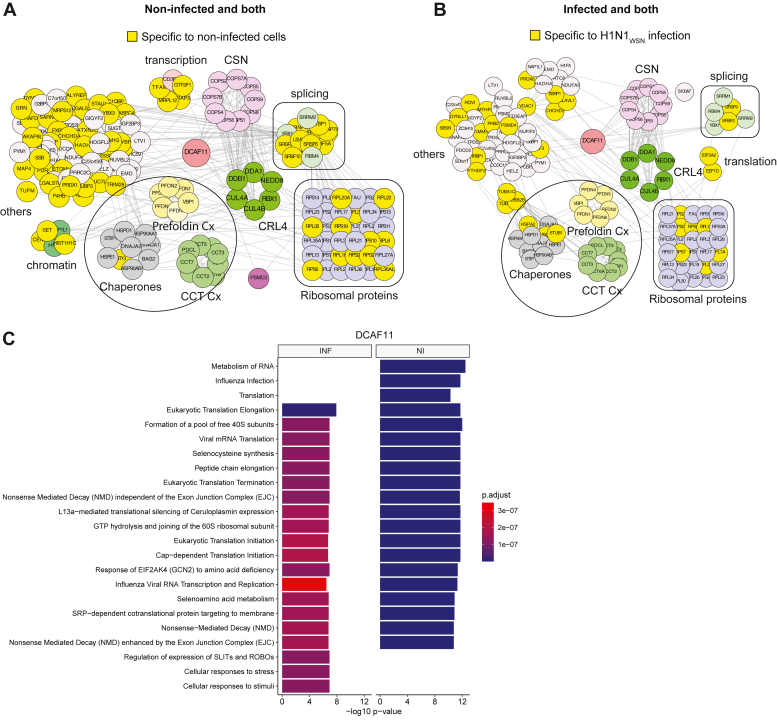
**Protein interaction network and functional assessment of DCAF11-associated proteins upon H1N1**_**WSN**_**infection**. *A*, proteins co-purified with DCAF11 in non-infected cells, corresponding to the combination of proteins associated with DCAF11 in both non-infected and infected conditions (both) and those found specifically in non-infected cells. *B*, proteins co-purified with DCAF11 in infected cells, correspond to the combination of proteins identified in both conditions and those only in infected cells. Clusters of proteins functionally enriched are highlighted in different colors. Proteins associated with DCAF11, specifically in non-infected (*A*), or infected (*B*) conditions, are depicted in *yellow*. *C*, gene ontology enrichment analysis of all significantly changing proteins using Benjamini-Hochberg correction analysis. HEK293 gene set from the Human Protein Atlas was used as a reference for background. INF, H1N1_WSN_ infected condition; NI = non-infected condition. All DCAF11-associated proteins are listed in Supplemental Table S2.

DCAF11 has been established to function as an SRF of CRL4 (42, 43). We identified 23 out of the 103 interactors of DCAF11 reported in BioGRID (https://thebiogrid.org/123251) (Supplemental Fig. S3). These interactors included components of CRL4 E3 ligase, the CRL4 regulator complex CSN, and the CCT complex (Supplemental Fig. S3), and we found their association with DCAF11 to be independent of IAV infection (Fig. 4, *A* and *B*). Similar to DDB1, DCAF11 is associated with protein chaperones and the prefoldin complex, which, in conjunction with the CCT complex, points to a connection between certain CRL4 ligases and protein folding. Additionally, DCAF11 engaged associations with transcription and chromatin factors, which were lost following infection. Conversely, distinct sets of ribosomal proteins and splicing factors were associated with DCAF11 in infected versus non-infected cells (Fig. 4, *A* and *B*). GO-term enrichment analysis pointed to the loss of factors regulating host translation, metabolism of RNA, and influenza A infection upon infection, while the DCAF11’s targeting upon infection affected cellular responses to stress and stimuli (Fig. 4*C*).

**Fig. 5:**
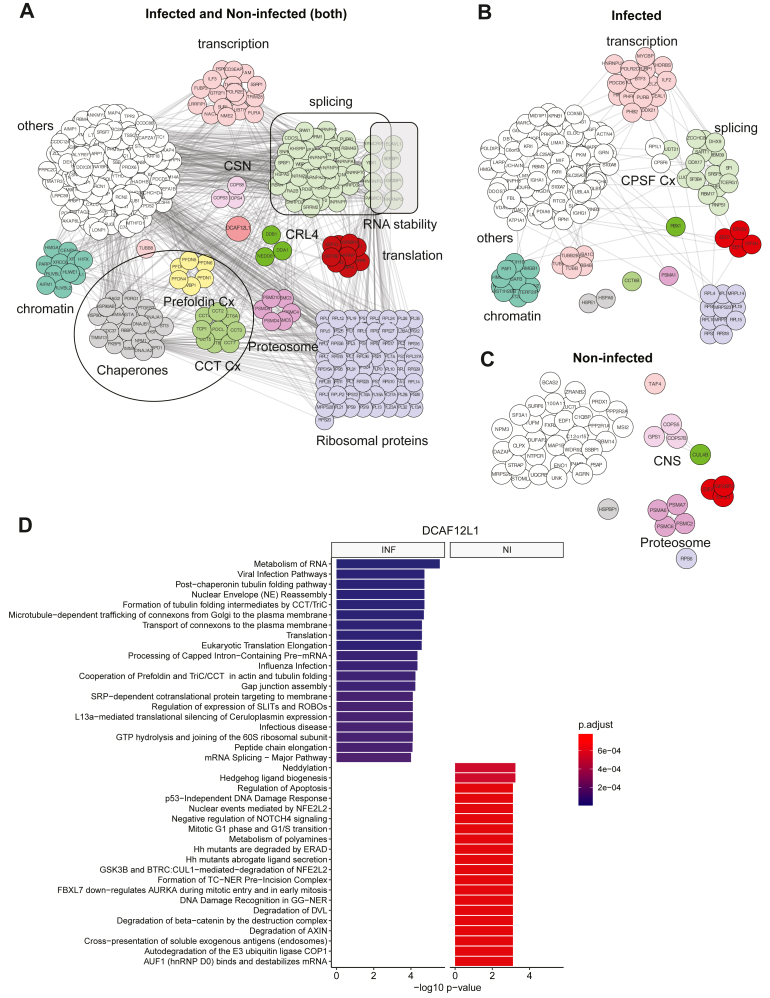
**Protein interaction network and functional assessment of DCAF12L1-associated proteins upon H1N1**_**WSN**_**infection**. *A*, proteins co-purified with DCAF12L1 in infected and non-infected cells (both). *B*, proteins associated with DCAF12L1, specifically in infected cells. *C*, proteins associated with DCAF12L1, specifically in non-infected cells. Clusters of proteins functionally enriched are highlighted in different colors. *D*, gene ontology enrichment analysis of all significantly changing proteins using Benjamini-Hochberg correction analysis. HEK293 gene set from the Human Protein Atlas was used as a reference for background. INF= H1N1_WSN_ infected condition, NI = non-infected condition. All DCAF12L1-associated proteins are listed in Supplemental Table S2.

Little is known about the function of DCAF12L1, which we nevertheless demonstrated to act as a *bona fide* SRF of CRL4, mediating ubiquitination of PB2 from IAV (18). Given the high number of cellular partners associated with DCAF12L1 (Fig. 2*D*), we categorized these association patterns based on three occurrences, i.e., present only in infected cells, only in non-infected cells, or in both conditions (Fig. 5, *A–C*). We detected the association of DCAF12L1 with CRL4 E3 ligase components and ribosomal proteins, with minor changes upon infection (Fig. 5, *A* and *B*). Protein chaperones, Prefoldin, and CCT complexes were among the consistent interactors of DCAF12L1, underscoring its significance in modulating protein folding, a fundamental process targeted by the three CRL4 factors studied. We also identified an association of DCAF12L1 with the proteasome and CSN complexes, with multiple subunits lost upon infection (Fig. 5, *A–C*). DCAF12L1 targeted proteins involved in mRNA splicing, transcription, and chromatin regulation, with increased associations during infection. Additionally, components of the Cleavage and Polyadenylation and Specificity Factor (CPSF), central components of the 3′ processing machinery for polyadenylated mRNAs, were recruited specifically upon infection (Fig. 5*B*). The top-enriched GO-terms targeted by DCAF12L1 associated factors upon infection include processes exploited by influenza virus, such as metabolism of RNA, RNA splicing and more precisely the processing of capped-intron-containing pre-mRNA which may be related to the influenza-specific process of cap snatching. In non-infected cells, DCAF12L1 showed a higher association with factors impacting UPS-related processes such as neddylation, proteasome, and protein degradation (Fig. 5*D*). Such GO-term enrichment supports our previous proposals that (i) DCAF12L1 might be the preferred SRF of CRL4s during infection, (ii) the CRL4s-mediated ubiquitination might be shifted toward non-proteolytic, proteasome independent ubiquitination under infection.

Our MS-based analysis of the CRL4’s associated factors upon IAV infection revealed a remodeling of their respective interactomes. Different binding profiles emerged, with the number of associated partners decreasing upon infection for DDB1 and DCAF11, while DCAF12L1 gained interactors. Our results are consistent with a modified repertoire of active CRL4 E3 ligases upon IAV infection, with those using DCAF12L1 as an SRF gaining a prevailing effect in CRL4-mediated ubiquitination that serves infection. Protein translation, RNA metabolism/processing, or splicing are among the main cellular processes impacted by CRL4’s rewiring upon infection, probably accounting for their intricate control over the course of the infection.

### N2H-Based Identification of Direct Targets of the CRL4s

**Fig. 6:**
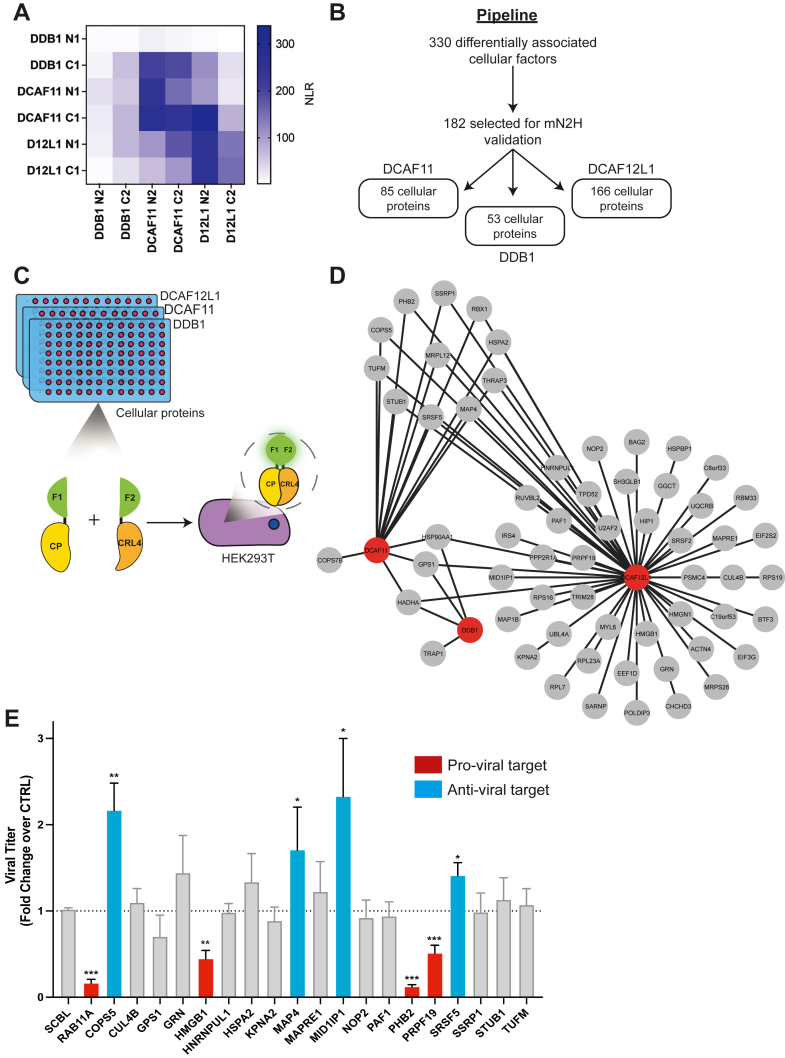
**Validation of CRL4-associated cellular targets using N2H assay and their role upon H1N1**_**WSN**_**infection**. *A*, schematic representation of the mN2H experimental approach. The cellular protein and CRL4s are fused with F1 and F2, respectively. *B*, selection pipeline of the CRL4s-associated cellular factors from our MS dataset for validation in mN2H. *C*, binary interactions between DDB1, DCAF11, and DCAF12L1. The optimal configuration was tested by combining all possible mN2 tagging configurations to detect the known interactions between DDB1 and DCAF11 or DCAF12L1. PPI was measured as a Normalized Luminescence Ratio (NLR) and represented as a heat map. *D*, direct interaction network between three CRL4s and their targets. CRL4 factors were fused with the F2 (C-terminal) fragment of the nanoluciferase using a C-terminal configuration for DDB1 (DDB1-C2), and the N-terminal configuration for DCAF11 and DCAF12L1 (N2-DCAF11 and N2-DCAF12L1). Prey nodes are represented in *grey*, and bait nodes are represented in *red*. *E*, A549-ACE2 cells were transfected with siRNA nontarget (NT) or siRNA targeting CRL4’s interactors for 48 h and infected with H1N1_WSN_ at a multiplicity of infection (moi) of 10^−3^ pfu/cell. Viral titers were determined by plaque-forming assay at 24 hpi. ∗*p*-value<0.05, ∗∗*p*-value<0.005, ∗∗∗*p*-value <0.001 by Mann-Whitney multiple comparison *t*-tests.

To explore the direct protein-protein interactions (PPIs) within the MS dataset, we implemented an orthogonal approach based on a mammalian split-nano luciferase assay known as N2H assay (35). Briefly, the N2H assay involves the co-expression of a protein pair in HEK293T cells, with each partner fused to a hemi-nanoluciferase fragment, enabling the reconstitution of an active nanoluciferase enzyme. The choice of hemi-luciferase tagging configurations for the protein pair influences PPI detection (35). Therefore, we first aimed to determine the optimal configuration for N2H assay for detecting direct PPIs involving the CRL4 factors. To this end, we examined the binary interactions between DDB1 and DCAF11 or DCAF12L1, previously reported to be part of the CRL4 E3 ubiquitin ligase complex (18) (Fig. 6*A* and Supplemental Table S4). The N2H signals were calculated as a Normalized Luminescence Ratio (NLR) for each protein pair to measure the level of interaction, factoring in background signals against a negative control (see methods). For DDB1, a C-terminal fusion nanoluciferase (DDB1-C1/2) proved more effective in detecting interactions with both SRFs. For DCAF11, equivalent N2H signals were generated against DDB1 with either N- or C- configuration (N1/2- or DCAF11-C1/2), while for DCAF12L1, the N-terminal fusion (N1/2-DCAF12L1) appeared more suitable for detecting PPI with DDB1. Our PPI matrix generated robust signals upon co-expression of DCAF11 or DCAF12L1 with themselves or between DCAF11-DCAF12L1 pairs, while no interactions were detected with negative control proteins, indicating a propensity for homo- and hetero-dimer formation in line with the presence of DWD domains.

To assess binary PPIs within the AP/MS dataset encompassing the three CRL4 factors, we selected 182 cellular proteins (CP) available in the human ORFeome collection (http://horfdb.dfci.harvard.edu/) for binary PPI testing in N2H assay. This selection was made from a total of 330 CP that were differentially associated with the CRL4 factors (Fig. 6*B* and Supplemental Table S2). All CPs were tagged with the F1 hemi-nanoluciferase at their N-terminus (N1-CP), and the CRL4 factors were fused to the F2 fragments in the appropriate configuration, i.e. DDB1-C2, N2-DCAF11 and N2-DCAF12L1 (Fig. 6*C*). N2H experiments were performed in two biological replicates, each with four technical replicates (Supplemental Fig. S4, *A–C* and Supplemental Table S5). The positive NLR values determined for DDB1/DCAF11, DCAF11/DDB1, and DCAF12L1/DDB1, were used as standards to define the threshold of positive PPIs.

Of the 53 DDB1-associated factors assessed in N2H, only 4 (8%) scored positive for interaction with DDB1, consisting of the COP9 signalosome subunit GPS1, the HSP90A chaperone protein, the mitochondrial chaperone TRAP1, and the HADHA enzyme of the mitochondrial beta-oxidation pathway (Supplemental Table S5). For DCAF11, 15 of the 85 factors tested (17%) were identified as direct interactors (Fig. 6*D* and Supplemental Table S5): 4 of the 23 infection-specific factors (17%), 5 of the 40 infection-free specific factors (12.5%), and 6 of the 22 invariant DCAF11 partners (27.3%). These invariant partners include UPS factors (RBX1) and regulators (COP9 signalosome subunits COPS5, COPS7B, and GPS1), all previously identified as DCAF11 interactors in BioGRID (https://thebiogrid.org/123251). The four infection-specific factors that scored positive are the STUB1 E3 ubiquitin ligase, which targets misfolded chaperone substrates toward proteasomal degradation, the HSPA2 chaperone, the SSRP component of the FACT complex regulating chromatin organization, and prohibitin 2 (PHB2), which acts, among others as a receptor for mitophagy. Additionally, positive interactors of DCAF11 that were lost upon infection encompass splicing factors THRAP3 and SRSF5, the microtubule-associated protein MAP4, mitochondrial ribosomal protein MRPL12, and the TUFM mitochondrial translation elongation factor.

Of 542 factors found associated with DCAF12L1 in AP-MS, 166 were tested in N2H assay, of which 58 (35%) exhibited direct binding to DCAF12L1 (Supplemental Table S5). Among these, 25 of the 73 infection-specific (34%) and 10 of the 26 infection-free specific factors (38.4%) are directly bound to DCAF12L1. Additionally, 23 factors maintained their association with DCAF12L1 independent of infection, including UPS factors and regulators, proteins involved in translation and folding, and RNA processing. Interestingly, eight of these factors also interacted with DCAF11, suggesting that both DCAFs are involved in the regulation of common cellular processes (Fig. 6*D*). In contrast, only 3 of the 25 factors binding to DCAF12L1 upon infection also interacted with DCAF11 (Supplemental Table S6), suggesting a rewiring specific to DCAF12L1, which primarily encompasses transcription factors and chromatin regulators.

### Exploration of the Functional Impact of the CRL4s Rewiring

To assess the functional relevance of our datasets, we first conducted literature mining to determine the role of 41 host proteins that differentially interacted with either DCAF11 or DCAF12L1 upon infection. Out of these, 19 proteins are reported as being involved in influenza virus infection (Supplemental Table S7). They all include factors binding to one of the SRFs (primarily DCAF12L1). To further assess the functional impact of CRL4’s rewiring, we performed siRNA-mediated silencing of 19 differentially targeted CRL4 partners in A549-ACE2 cells, followed by infection with the H1N1_WSN_ strain. RAB11A was taken as a positive control as its depletion was previously reported to affect influenza virus production (44). The knockdown efficacy of these factors was measured by RT-qPCR (Supplemental Fig. S5). The silencing of 7 out of the 19 explored rewired CRL4 partners had an impact on infection compared to non-targeting siRNA (SCBL) (Fig. 6*E*), supporting their involvement in the viral life cycle. Among them, 3 emerge as pro-viral factors, whose depletion affects the virus production: HMGB1, PHB2, and PRPF19, corroborating their contribution to IAV infection (45, 46, 47). By contrast, silencing of the other 4 siRNAs led to increased viral production: COPS5, MAP4, MID1IP1, and SRSF5, indicative of antiviral factors whose depletion benefits to the viral cycle (48, 49, 50, 51) (Fig. 6*E* and Supplemental Table S8). Thus, the differential binding of these factors to SRFs upon infection likely contributes to modulating the influenza virus life cycle.

## Discussion

The significance of the UPS in virus replication and pathogenesis is underscored by its involvement in the ubiquitination of viral proteins or modifications in the host proteome ubiquitination. Despite multiple documented UPS-mediated modifications of IAV viral proteins, the impact of infection on host proteome regulation through the UPS has remained largely unexplored. To address this gap, we aimed to investigate how IAV infection impacts the host proteome binding repertoire of two specific E3 ubiquitin ligase complexes, namely CRL4^DCAF11^ and CRL4^DCAF12L1^. Previously, we reported that these multicomponent E3 ubiquitin ligases promote IAV infection through non-degradative ubiquitination of the viral PB2 protein. Using AP-MS proteomics, we identified changes in DDB1, DCAF11, and DCAF12L1-associated proteins in both non-infected and H1N1_WSN_ virus-infected human HEK293 cells. Our analyses revealed substantial alterations in the associated proteins of CRL4 factors upon infection, uncovering the rewiring of functionally relevant E3 ubiquitin ligases during IAV infection.

The roles of DDB1 and DCAF11 as CRL4 components are well-documented (40, 41, 42). We successfully identified several of their known interacting partners, validating the robustness of our experimental AP-MS datasets. To our knowledge, this study provides the first comprehensive report on the DCAF12L1 interactome, revealing its association with other CRL4s components, supporting our prior finding that it can function as an SRF of CRL4 E3 ubiquitin ligases (18). All three CRL4 factors were associated with the proteasome complex, responsible for the degradation of ubiquitinated proteins. Interestingly, such association decreased upon infection for DDB1 and DCAF12L1, suggesting a shift towards non-proteolytic ubiquitination, consistent with our previous findings on PB2 ubiquitination (18). Components of the de-neddylation CSN complex, a negative regulator of CRLs (52), were also associated with the three CRL4s factors. Such associations were already reported with some DCAF proteins, including DCAF11 (53), and could mediate the sequestration of pre-assembled CRLs. Chaperones, including the prefoldin and/or the TriC/CCT complexes, emerged as partners of the three CLR4s factors, with their association to DDB1 being decreased upon infection while remaining constant with both SRFs. Remarkably, the PB2 subunit of IAV polymerase co-purified with chaperones, including Hsp90 and members of the CCT complex in an infection context (41, 54), highlighting the importance of protein folding in influenza infection, and implicating CRL4 E3 ligases in this process.

At a global level, the remodeling of CRL4 factors upon IAV infection exhibited opposite trends, with a loss of DDB1- and DCAF11-associated factors and a gain of DCAF12L1-associated factors, suggesting a shift toward CRL4^DCAF12L1^ E3 ubiquitin ligases upon infection. Infection-induced alterations in the binding profiles of DCAF11 and DCAF12L1 targeted common cellular processes, including translation, RNA metabolism, and transcription, all intricately regulated through ubiquitination. Functional specificities also emerged for the two SRFs, with, for example, pathways related to cellular response to stress or stimuli being specifically targeted by DCAF11 upon infection, while multiple mechanisms linked to the trafficking of proteins to membranes are targeted by DCAF12L1. Moreover, multiple mechanisms related to ubiquitination-induced degradation of proteins associated with DCAF12L1 are lost upon infection, in line with the observed release of proteasome from DCAF12L1.

Our AP-MS datasets illustrate the co-complexes associated with the CRL4s factors. To increase the precision of their rewiring induced by IAV infection, we probed AP-MS datasets for direct interactions using a split nanoluciferase-based N2H assay (35). Overall, our data highlight a connection between CRL4s and mitochondrial proteins: the HADHA mitochondrial enzyme is bound by the three CRL4 factors, both SRFs interact with MRPL12, TUFM, and PHB2, and the mitochondrial chaperone TRAP1 interacts with DDB1. In line with this, CRL4 E3 ligases recently emerged as critical for the regulation of mitochondrial structure and function (55). Moreover, a fraction of the PB2 protein from human IAV localizes in mitochondria, where it may contribute to preserving mitochondria functions (56, 57). It is possible that CRL4-mediated ubiquitination of mitochondrial proteins participates in maintaining mitochondria integrity, which nevertheless remains to be addressed. Additionally, three RNA polymerase II binding proteins, PAF1, PRPF19, and TCERG1I, were shown to interact with DCAF12L1. Such interactions could mediate the ubiquitination and degradation of RNA polymerase II via the proteasome pathway occurring at late stages of infection (58), and CRL4 ^DCAF12L1^ E3 ligases may be involved in such heretofore undiscovered activity. As SRF, DCAF proteins serve to recruit target proteins for CRL4-mediated ubiquitination. The distinct PPI profiles of DCAF11 and DCAF12L1 likely reflect their different roles toward ubiquitination and potentially other cellular activities.

To assess the functional relevance of the rewired proteins, we proceeded to a literature search for each CRL4 interactor and identified 19 out of 41 proteins with a reported role in IAV infection. We assessed the impact of siRNA-mediated depletion of these 19 targets on productive infection in A549 cells and confirmed the role of 7 of them. In addition, 3 of the CRL4’s interactors reduced the production of viral particles, suggesting a pro-viral role. These include HMGB1, PHB2, and PRPF19. In contrast, the silencing of 4 of the CRL4s interactors favored infection, suggesting an antiviral role, and restrictive activity. These include COPS5, MAP4, MID1IP1 and SRSF5. All these factors had been reported previously as involved in influenza virus infection (Supplemental Table S7), and we confirmed here their involvement in the viral life cycle, thus providing evidence of the implication of the rewiring of the CRL4s in influenza virus infection.

The unbiased global proteomics presented here mapped the landscape of interaction partners of two CRL4^DCAF11^ and CRL4^DCAF12L1^ complexes of known relevance for IAV infection and uncovered their virus-induced rewiring. Alterations in their interaction profile upon IAV infection might contribute to the proteome remodeling triggered by IAV infection and participate in shaping a cellular environment conducive to viral infection. As such, it can ultimately be leveraged for the development of antiviral therapeutic strategies against influenza virus infection.

## Data Availability

Annotated data related to Supplemental Table S1 are available at: https://msviewer.ucsf.edu/prospector/cgi-bin/mssearch.cgi?report_title=MS-Viewer&search_key=lhmasg5cia&search_name=msviewer

## Supplemental data

This article contains supplemental data (12, 45, 46, 47, 48, 49, 50, 51, 59, 60, 61, 62, 63, 64, 65, 66, 67).

## Conflict of interest

The authors declare that they have no conflicts of interest with the contents of this article.

## Supplementary Data

Supplemental Fig. S1 Selection of single clones for Strep-tagged CRL4 stable cells and quality control of Strep pull-down. *A*, HEK-293 cell lines stably expressing Strep-tagged fused DDB1, DCAF11, DCAF12L1 or mCherry (control) were harvested. The expression level of Strep-tag, DDB1, and GAPDH were detected in whole cell lysates by western blot using corresponding antibodies. ∗denotes the cell clones chosen and proceeded for AP/MS, ˆ indicates a lane with a cherry-expressing cell clone loaded among the DCAF11-expressing ones. *B*, mRNA levels of DDB1, DCAF11 and DCAF12L1 in HEK-293 cells stably expressing Strep-tagged CRL4s compared to HEK-293 cells measured by RT-qPCR. *C*, Control of Strep affinity purification and purified proteins elution from lysates of the indicated cells. Bands corresponding to each Strep-CRL4 factor and mCherry are indicated with *arrows*.

Supplemental Fig. S2 Quality control metrics and correlation between biological replicates in H1N1_WSN_ infected and non-infected cells. *A*, Barplot of missing values in each sample calculated from the total number of identified proteins. *B*, Pearson correlation matrix between all samples using shades of *blue*. *Darker blues* indicate higher correlation values. *C*, Hierarchical clustering of samples from Pearson correlation values. *D*, Distributions of log2 intensities of peptides found in all samples before normalization by median centering in each condition. *E* and *F*, Correlation matrix of the three biological replicates of HEK-293 cell lines stably expressing Strep-DCAF11 or Strep-DCAF12L1 *versus* Strep-Cherry controls in H1N1WSN infected cells (*E*) or non-infected cells (*F*).

Supplemental Fig. S3 Overlap of MS data of DCAF11 with known interactors. Interaction network of the DCAF11. Known partners detected in our MS analysis and retrieved from BioGRID are highlighted in *orange*.

Supplemental Fig. S4 Correlation matrix of N2H signals across replicates. *A*–*C*, Scatter plot of Raw Luminescence Unit (RLU) for each replicate (two biological replicates each with four technical replicates) calculated and annotated using Pearson correlation coefficient on each of the CRL4-factors DDB1 (*A*), and DCAF11 (*B*), DCAF12L1 (*C*). The raw data for each replicate associated with the CRL4s are listed in Supplemental Table S5.

Supplemental Fig. S5 Confirmation of siRNA-mediated silencing of identified CRL4 interactors. A549-ACE2 cells were transfected with siRNA nontarget (NT) or siRNA targeting CRL4’s interactors. 48 h post-transfection cells were lysed, and mRNA expression level of the corresponding targets was measured by RT-qPCR.

Supplemental Table S1 Affinity Purification-Mass Spectrometry: identified proteins and statistical analysis.

Supplemental Table S2 List of Mass-Spectrometry identified interactors for each of the CRL4 factors and their detection status in AP-MS.

Supplemental Table S3 GO-term enrichment analysis results.

Supplemental Table S4 Raw data (RLU) from the CLR4 mN2H experiment.

Supplemental Table S5 Raw data (RLU) value of each of the two biological replicates and NLR analysis of the mN2H validation of the cellular factors for each CRL4 factor.

Supplemental Table S6 List of common CRL4 interactors identified positively in AP-MS (Tab A), and positively in mN2H (Tab B).

Supplemental Table S7 List of cellular factors associated with DCAF11 and DCAF12L1 or both that are selected for functional assessment in IAV infection. The experiment was done in two biological replicates each with at least 3 technical replicates.

Supplemental Table S8 Raw data for each functional validation of CRL4s interactors for each replicate and statistical analysis. The experiment was done in two biological replicates each with at least 3 technical replicates.
