# Supplementary figures and images for: Profiling Cullin4-E3 Ligases Interactomes and Their Rewiring in Influenza A Virus Infection
Source: Mol Cell Proteomics. 2024 Oct 9;23(11):100856. doi: 10.1016/j.mcpro.2024.100856 (PMC11609542; doi:10.1016/j.mcpro.2024.100856)

# Supplemental Fig. S1

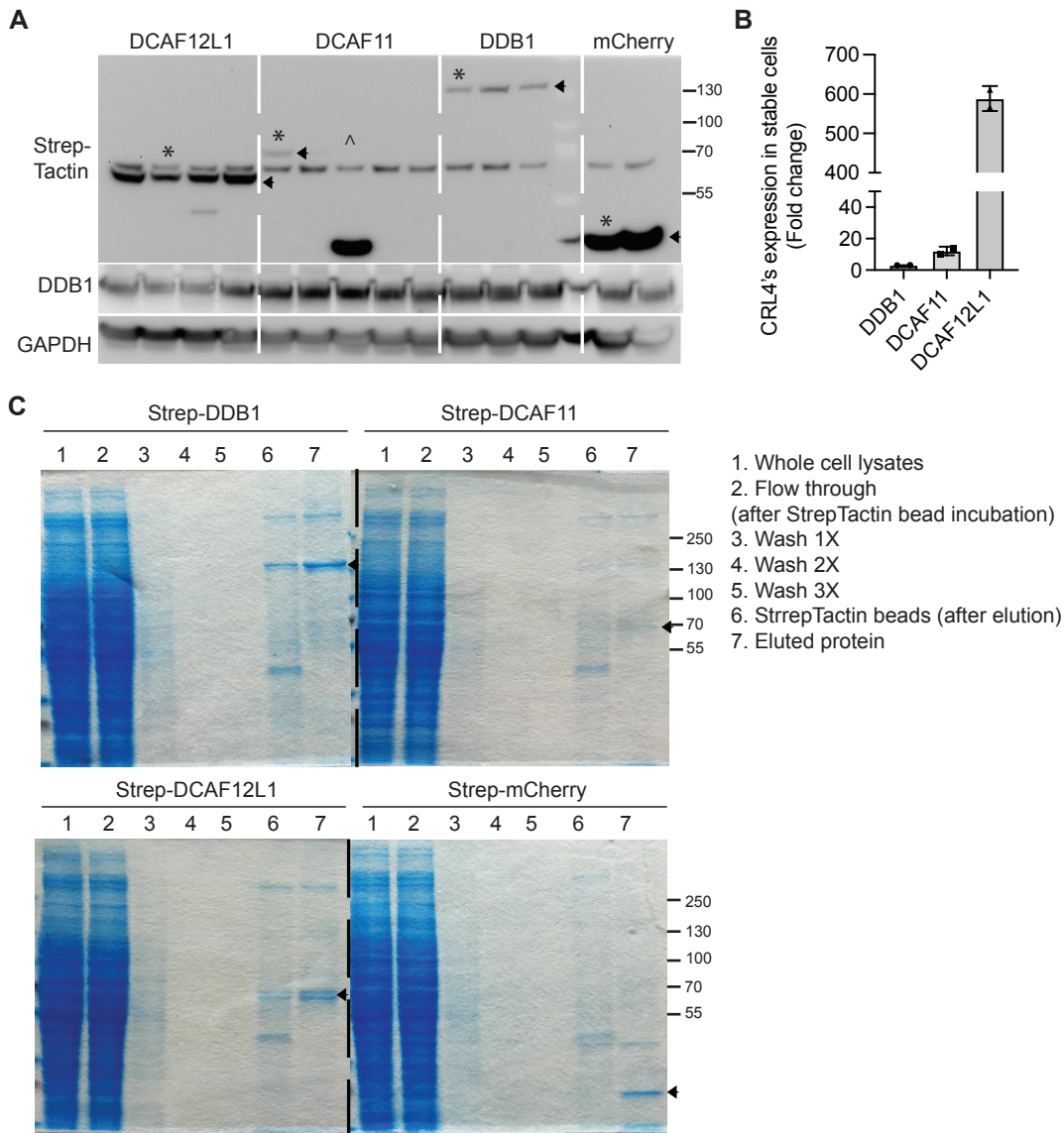

Supplement: Supplemental Fig. S1 — Selection of single clones for Strep-tagged CRL4 stable cells and quality control of Strep pull-down. A, HEK-293 cell lines stably expressing Strep-tagged fused DDB1, DCAF11, DCAF12L1 or mCherry (control) were harvested. The expression level of Strep-tag, DDB1, and GAPDH were detected in whole cell lysates by western blot using corresponding antibodies. ∗denotes the cell clones chosen and proceeded for AP/MS, ˆ indicates a lane with a cherry-expressing cell clone loaded among the DCAF11-expressing ones. B, mRNA levels of DDB1, DCAF11 and DCAF12L1 in HEK-293 cells stably expressing Strep-tagged CRL4s compared to HEK-293 cells measured by RT-qPCR. C, Control of Strep affinity purification and purified proteins elution from lysates of the indicated cells. Bands corresponding to each Strep-CRL4 factor and mCherry are indicated with arrows. [file mmc1.pdf]

## Supplemental Fig. S2

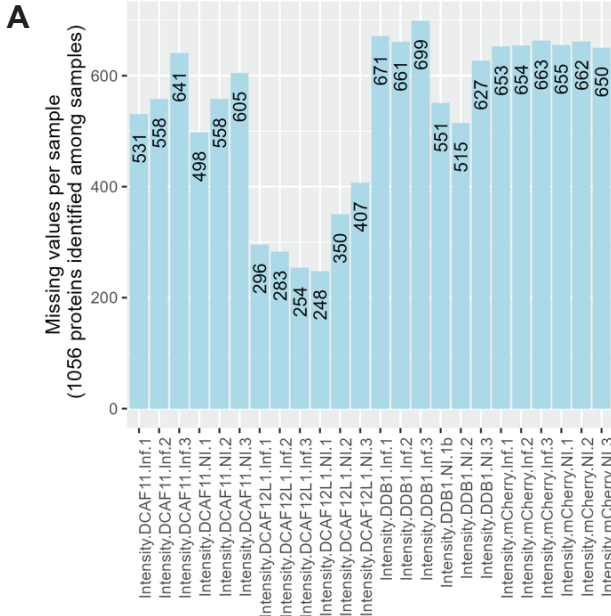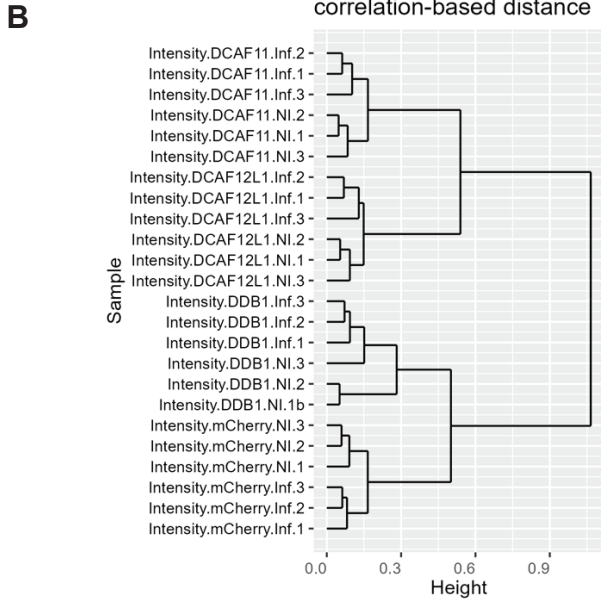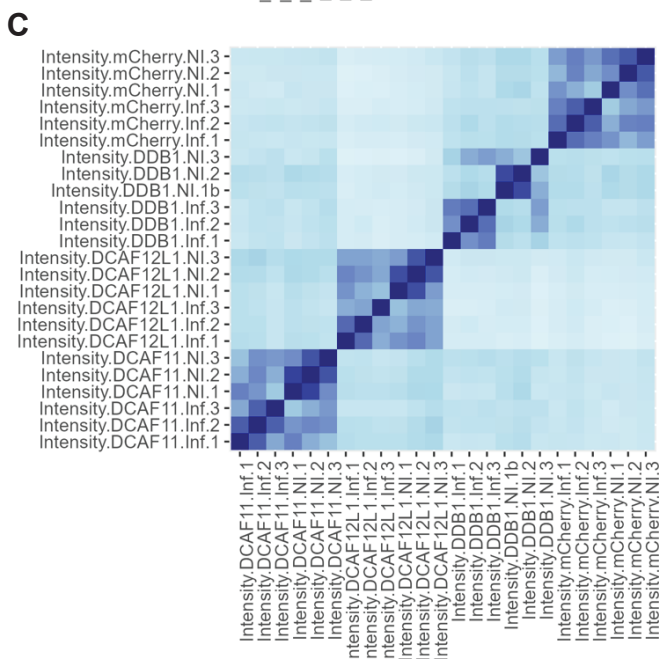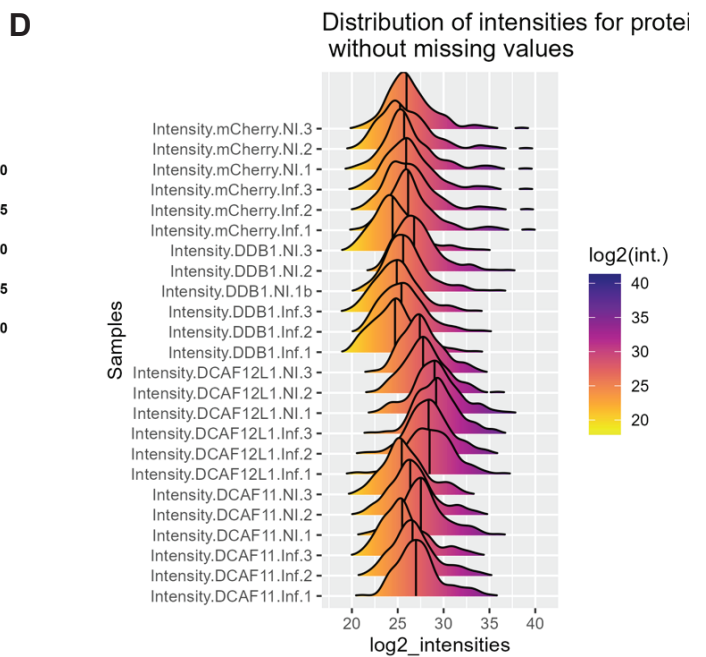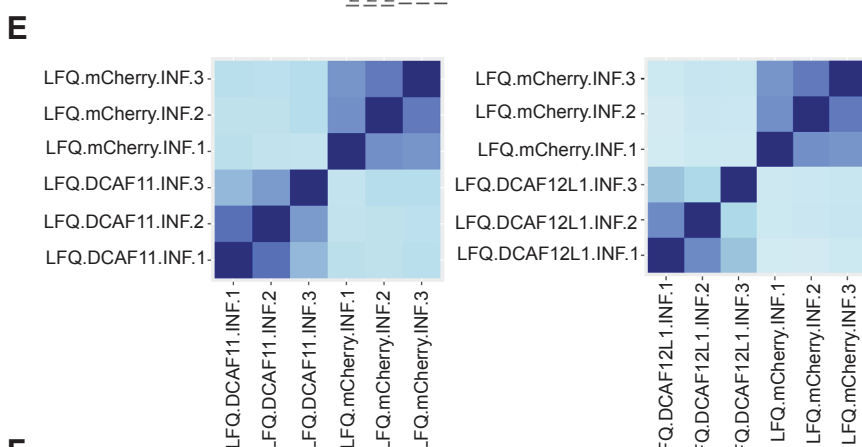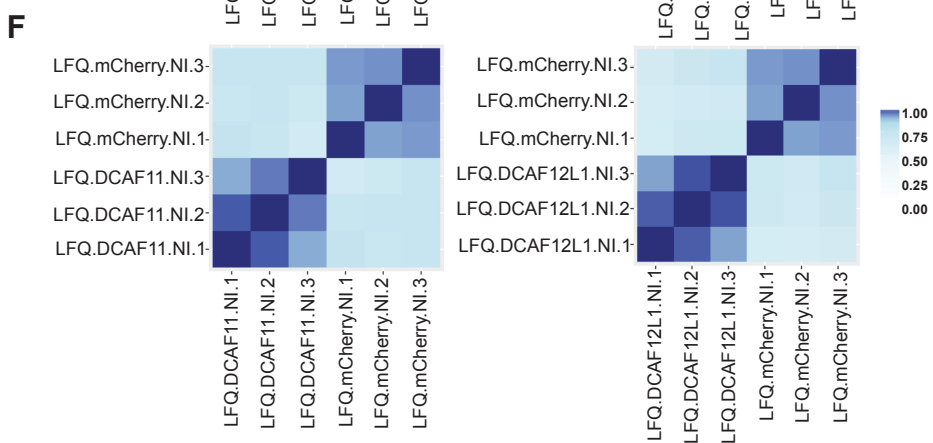

Supplement: Supplemental Fig. S2 — Quality control metrics and correlation between biological replicates in H1N1WSN infected and non-infected cells. A, Barplot of missing values in each sample calculated from the total number of identified proteins. B, Pearson correlation matrix between all samples using shades of blue. Darker blues indicate higher correlation values. C, Hierarchical clustering of samples from Pearson correlation values. D, Distributions of log2 intensities of peptides found in all samples before normalization by median centering in each condition. E and F, Correlation matrix of the three biological replicates of HEK-293 cell lines stably expressing Strep-DCAF11 or Strep-DCAF12L1 versus Strep-Cherry controls in H1N1WSN infected cells (E) or non-infected cells (F). [file mmc2.pdf]

Supplementary Figure 3

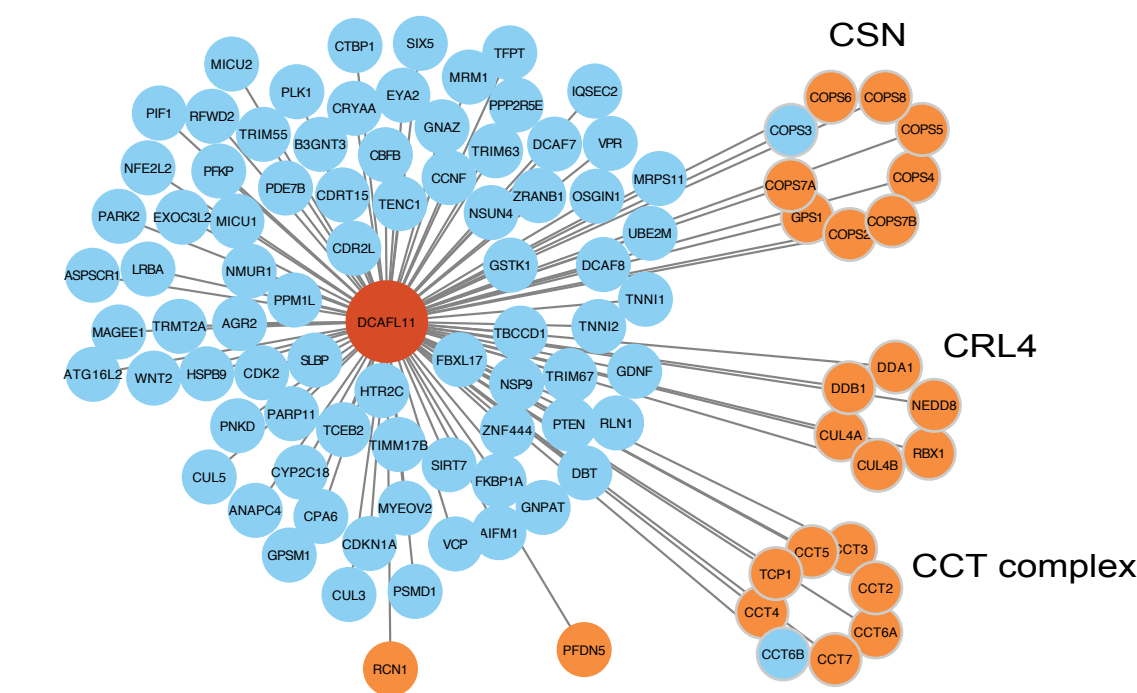

Supplement: Supplemental Fig. S3 — Overlap of MS data of DCAF11 with known interactors. Interaction network of the DCAF11. Known partners detected in our MS analysis and retrieved from BioGRID are highlighted in orange. [file mmc3.pdf]

# Supplemental Fig. S4

**A**

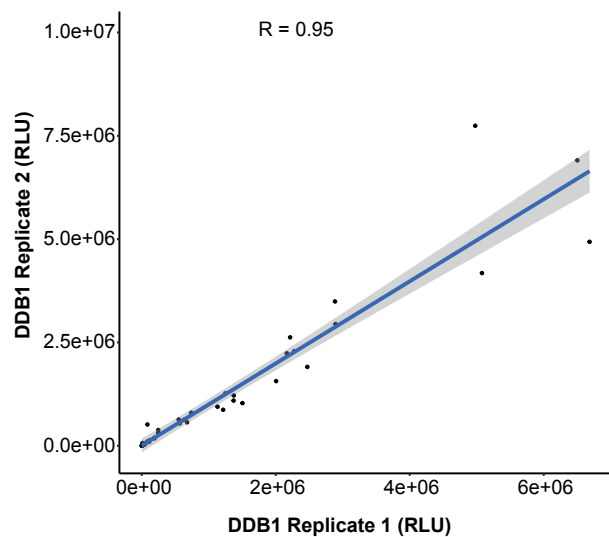

**B**

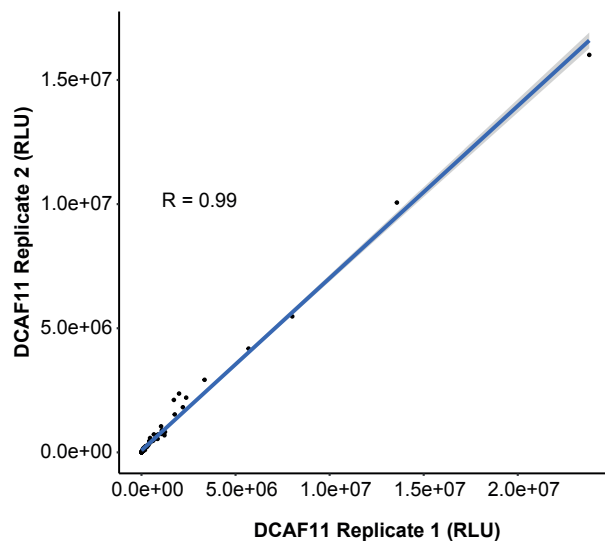

**C**

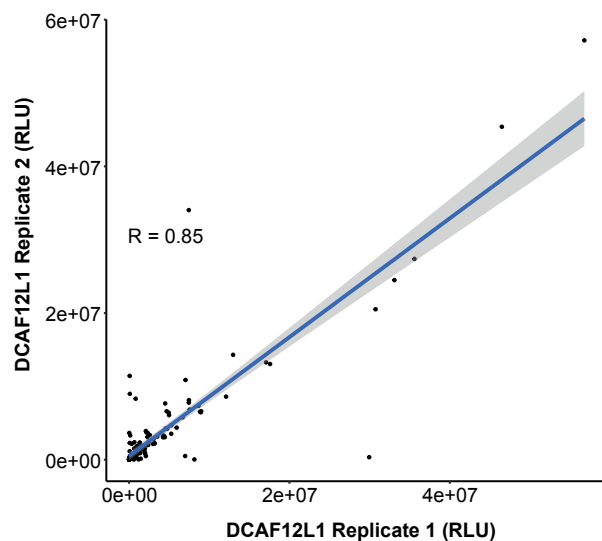

Supplement: Supplemental Fig. S4 — Correlation matrix of N2H signals across replicates. A–C, Scatter plot of Raw Luminescence Unit (RLU) for each replicate (two biological replicates each with four technical replicates) calculated and annotated using Pearson correlation coefficient on each of the CRL4-factors DDB1 (A), and DCAF11 (B), DCAF12L1 (C). The raw data for each replicate associated with the CRL4s are listed in Supplemental Table S5. [file mmc4.pdf]

Supplemental Fig. S5

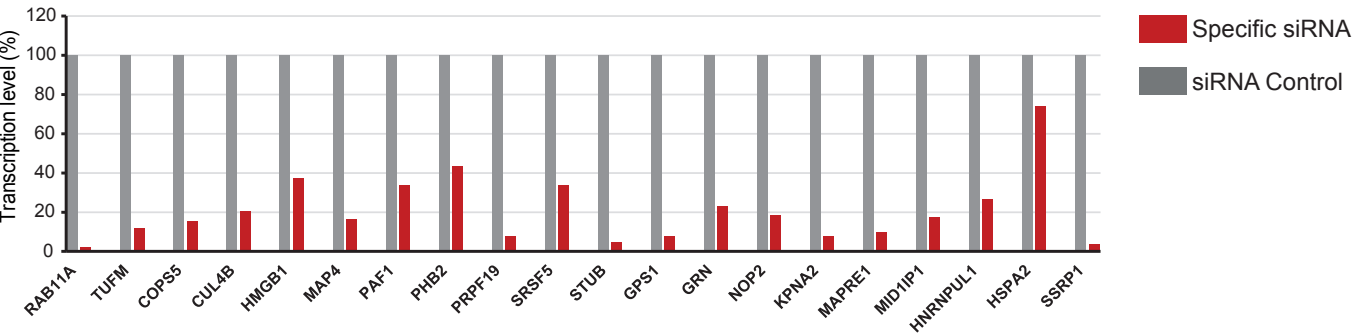

Supplement: Supplemental Fig. S5 — Confirmation of siRNA-mediated silencing of identified CRL4 interactors. A549-ACE2 cells were transfected with siRNA nontarget (NT) or siRNA targeting CRL4’s interactors. 48 h post-transfection cells were lysed, and mRNA expression level of the corresponding targets was measured by RT-qPCR. [file mmc5.pdf]
